# From the Ground Up: A Multidisciplinary Approach to Past Fertility and Population Narratives

**DOI:** 10.1007/s12110-023-09459-x

**Published:** 2023-09-19

**Authors:** Clare McFadden

**Affiliations:** 1 Max Planck Institute for Evolutionary Anthropology, 04103 Leipzig, Germany; 2 School of Archaeology and Anthropology, Australian National University, Acton, ACT 2601 Australia

**Keywords:** Fertility, Past Populations, Population Dynamics, Paleodemography, Structural Equation Model

## Abstract

Population dynamics form a crucial component of human narratives in the past. Population responses and adaptations not only tell us about the human past but also offer insights into the present and future. Though an area of substantial interest, it is also one of often limited evidence. As such, traditional techniques from demography and anthropology must be adapted considerably to accommodate the available archaeological and ethnohistoric data and an appropriate inferential framework must be applied. In this article, I propose a ground-up, multidisciplinary approach to the study of past population dynamics. Specifically, I develop an empirically informed path diagram based on modern fertility interactions and sources of past environmental, sociocultural, and biological evidence to guide high-resolution case studies. The proposed approach is dynamic and can evolve in response to data inputs as case studies are undertaken. In application, this approach will create new knowledge of past population processes which can greatly enhance our presently limited knowledge of high-frequency, small-scale demographic fluctuations, as well as contribute to our broader understanding of significant population disturbances and change throughout human history.

The human narrative through deep time is one of great interest and fragmented evidence. Using the preserved remnants of human activity and humans themselves, we seek to reconstruct all aspects of life for individuals, communities, and populations. Populations and their dynamics undoubtedly constitute a core component of human narratives. Their highs and lows, responses and adaptations, not only tell us about the human past but offer insights into our own present and future. Many of us recognize that population dynamics are not disconnected from communities or individuals but, rather, that individuals and communities drive fluctuations in population dynamics and feel the weight of their impacts. It is interesting, then, to observe that much of the paleodemographic literature to date is focused on somewhat loose definitions of both *population* and *dynamics*, with an overwhelming focus on regionally represented populations (often on a continental or at least a national basis) and major shifts in population growth or decline. In such research, we have all but removed from the study of past populations the individual, the community, and the discrete demographic variables that influence growth. If we are to improve our understanding of the factors influencing population changes, as well as the nuances of adaptive and maladaptive population responses, and contribute to multiscalar human narratives, we must examine the evidence more closely and through a theoretically informed lens.

Human remnants can take many forms of evidence, and a vast array of methodologies can be applied to their analysis for the purpose of paleodemographic research. Preserved human remains can be analyzed using ancient DNA and skeletal analyses to build a biological profile for each individual (including their sex, age, stature, evidence of pathology, mobility, and ancestry). Extrapolated to the population level, genetic studies can use a combination of ancient and modern DNA to identify isolation, migration, and population size estimates (Loog, 2021). Biological profile data can be used to evaluate the age-at-death distribution of the population, which provides a number of population insights by proxy, including fertility, birth, maternal mortality, and intrinsic population increase rates (Bocquet-Appel & Masset, 1982; McFadden & Oxenham, 2018a, 2018b, 2019). The frequency or concentration of radiocarbon dates, when radiocarbon represents evidence of human activity, can also be used to evaluate relative fluctuations in human activity and, by proxy, population growth (developed initially by Rick [1987] but with significant advancement since then; e.g., DiNapoli et al., 2021). A range of other techniques, such as ethnographically informed estimates of house capacity applied to preserved settlement structures (e.g., Porčić & Nikolić, 2016), have also been used to estimate population dynamics in the past.

Much of the research to date uses regional analyses of large geographic areas to address big questions about past population responses, such as the impacts of agriculture (Bettinger, 2016; Goldberg et al., 2016; Oh et al., 2017; Pardo-Gordo & Carvalho, 2020; Timpson et al., 2014; Zahid et al., 2016). But this leaves a wealth of information to be uncovered about smaller, shorter term fluctuations in population dynamics, which would be felt by members of the population no less, and perhaps even more, than longer temporal span changes. As argued by Boone (2002), the observed long-term growth rates of close to 0% (for example, see Zahid et al., 2016) are a product of an averaging effect of peaks and crashes throughout human history (Page & French, 2020); therefore, our focus on broader trends leaves a great number of population narratives to be explored. Furthermore, many of the variables that we might expect to interact with and influence population dynamics occur on a spatially local scale, such as localized ecology, culture, and technology. And, since populations are static in neither space nor time, more localized studies give us better capacity to accurately represent population units in a meaningful way. With the disparate nature of past demographic evidence in mind, what hope do we have of undertaking such high-resolution, strongly contextualized, and ultimately nuanced studies of past populations? In this paper, I advocate for a multidisciplinary and empirically informed approach to studies of past populations, which uses a combination of past and modern evidence to estimate and enlighten paleodemographic narratives.

The approach proposed here has two core aims: firstly, to guide high-resolution case studies of population responses (specifically, fertility) which provide a deeper understanding and more accurate representation of the complex interplay of causal and resultant factors, creating nuanced population narratives, and secondly, to build a model that can estimate population responses in the absence (or scarcity) of population proxy data using a combination of contextual variables influencing the population. As a first step to achieve these aims, I propose a path diagram. Path analysis identifies the variables that influence an outcome (in this case, fertility rate) through multiple causal pathways, accommodating both direct and indirect effects (Lleras, 2005). The path diagram is a graphic representation of the relationship between variables, which are set out in outlined shapes, with arrows indicating directional relationships and lines (or wires) indicating undirected relationships. Latent variables are indicated by text or differentiated by shape (e.g., box for observable variables and oval for latent variables). A great benefit of using path analysis for studies of past populations is that it requires clearly articulated and logically set out causal pathways, which in the case of fertility would include consideration of how indirect (such as subsistence change) and direct (such as decreased age at menarche) causes relate to one another and to fertility. This process can, and arguably should, be resource intensive, to ensure the model is appropriately grounded by theory and empirical evidence.

The model allows for exogenous variables, which influence the outcome but are not influenced by other factors in the model, and endogenous variables, which influence the outcome and may also be influenced by factors in the model (Lleras, 2005). This can, at least partially, accommodate feedback loops that are typical of demographic processes. Path analysis is reasonably commonplace in demographic research in modern populations (Caudell & Quinlan, 2012; Jasienska & Ellison, 2004; Snopkowski et al., 2016) but has yet to be applied to past demographic data. A large part of this paper is dedicated to the development of a path diagram, which is not only well considered and grounded, but also reflects the opportunities and limitations of the data available for studies of past populations. As such, it seeks to describe the types of variables we may expect to interact with fertility in past populations broadly in a range of environmental and social contexts, based on modern empirical evidence, and identify example archaeological and historical proxies for these.

A path diagram can subsequently be used to form the relational model component of a structural equation model approach. This second phase of the *ground-up* approach progresses the empirically informed path diagram to a practically applicable analysis of paleodemographic and associated data. By analyzing data collected through path-guided case studies, the strength of relationships between indirect and direct effects and fertility outcomes can be evaluated. A major advantage of structural equation modeling is that it can accommodate nonlinearities, where variables may be both cause and effect (Bollen & Noble, 2011; Lleras, 2005). A further benefit is that unobservable variables, known as latent variables, can be built into the model.

The focus of this paper is the initial stage of developing a structural equation model approach (known as model specification) for use in paleodemographic studies. I have provided a very basic path diagram on this basis as a foundational model for developing spatially and temporally localized models. These models can then be progressed to structural equation modeling and can guide highly contextualized investigations into multidimensional fertility interactions. Though it is beyond the scope of this paper to undertake such a highly contextualized case study of the many factors known to interact with fertility (which, as previously noted, should be resource intensive if it is to be comprehensive and accurate), a simplified example of adapting the model to a specific regional context (in this case, adapting the broad path diagram to a focus on a number of Pacific Island nations) is provided to demonstrate how to operationalize this approach.

## The Foundations of a Ground-Up Approach

If we are to gain a deeper understanding of fertility in past populations, we must examine the available empirical evidence for its relationship with other aspects of human experience, ecology, and environment, with reference to research from across the globe. Variables that may interact with fertility are examined under three broad categories:

1. energetic, physiological, and environmental resources and stress
2. mortality and anthropogenic and natural disasters
3. culture and agency

These are, notably, a combination of proximate and background determinants. Proximate determinants are the variables that directly influence fertility (e.g., age at menarche directly influences the length of the reproductive lifespan), whereas background determinants are factors that affect fertility via a direct determinant (e.g., energetic expenditure may influence age at menarche). Bongaarts (1978) set out the proximate determinants of fertility as the proportion of married women, contraceptive use, induced abortion, lactational amenorrhea, frequency of intercourse, sterility, spontaneous abortion, and reproductive lifespan. A later iteration by Wood (1994) offered a dynamic model with greater attention to biological, but still including behavioral, mechanisms. The variables in Wood’s (1994) model are divided into exposures (exposure to conditions for conception) and susceptibilities (the probability of conception given exposure). These include the ages at which menarche, menopause, sexual activity, and onset of pathological sterility occur; length of the ovarian cycle and occurrence of ovulation; duration of fertile period; insemination; pregnancy loss; the length of infecundability following pregnancy loss, of the gestation period, and of lactational amenorrhea. Wood’s (1994) model is arguably preferred over that of Bongaarts (1978, 2015) (see Vitzthum, 2020, for discussion); however, in application to past populations, any such model contains a large number of variables that are unobservable, and therefore only accessible through background proxies (if accessible at all).

Proximate determinants can vary in weighting among modern populations on a cultural and spatial (for example, the studies by Chola & Michelo, 2016, in Zambia and Majumder & Ram, 2015, in Southeast Asia), and genetic (see review by Kohler et al., 2006, which found genetic variance affects fertility outcomes directly and indirectly) basis, and therefore it is likely that they differ through time too. Cultural and demographic contexts are key to understanding which determinants may be relevant. In natural fertility populations, where birth control is not practiced, physiological factors and marriage customs may be anticipated to have a significantly greater impact on fertility outcomes (Bongaarts & Potter, 1983; Chola & Michelo, 2016; Wiley, 1998). In contrast, controlled fertility populations use abstinence and contraception in response to a variety of background factors (Bongaarts & Potter, 1983) and may therefore require a more complex model. Further, the status of a population with respect to demographic transition, when populations shift from high to low births and mortality, can have major implications for the degree to which variables influence fertility, while also varying within different cultural contexts (Colleran, 2016; Snopkowski et al., 2016). Context is demonstrably very important when considering proximate and background determinants of fertility.

Although many variables arguably belong to more than a single category, either by their nature or by the way they relate to other variables, these categories can offer a pragmatic guide to investigations into fertility in the past. It is also pertinent to all categories that individual and thus population responses to extrinsic and intrinsic factors may be adaptive or maladaptive. This has been commonly neglected in the discourse on past population dynamics but may explain dissimilarities in population dynamics within similar contexts. Though not exhaustive due to the vast range of variables that may influence or be influenced by fertility, the following literature review touches on a range of fertility interactions. The literature review is intended to encourage researchers to think about the complexity of fertility interactions, consider the realistic expectations of what we may learn about fertility in the past, and keep an open mind as to what variables and associated archaeological evidence may be relevant in the study of past fertility. This sets the foundations for path analysis and structural equation modeling of fertility in past populations. Researchers should consider how the determinants discussed in the literature review, which are largely background determinants, may influence fertility via the range of proximate determinants outlined by Wood (1994). Additionally, the complex and sometimes seemingly contradictory nature of the literature touched on in this review highlights the importance of transdisciplinary expert input into such research.

## Energetic, Physiological, and Environmental Resources and Expenditure

Fertility in past populations has largely been considered a product of parent biology and physiology, and it has commonly been implied that behavior and agency have a negligible effect. The physical capacity of the parents to conceive and the mother to carry a child to full term are clearly extremely important to individual and population fertility. Yet determining and extricating the biological factors that influence fertility has proven highly complex. Energetic resources (e.g., food) and stress (e.g., physical labor) are of great interest due to their relationship to age at menarche and, in sexually mature women, their impact upon sexual hormones and thus reproductive cycles. In studies of past fertility, agriculture has commonly been hypothesized to increase fertility since it provides greater and more stable energetic resources and decreases mobility-related expenditure (Hassan & Sengel, 1973; Sussman, 1972). But modern studies of energetic resources (or lack thereof) report the theoretical impact on fertility is rarely straightforward in empirical studies, except in extreme cases such as famine (Bongaarts, 1980). Both very low and very high calorie intake can decrease fertility (Panth et al., 2018; Silvestris et al., 2019); therefore, the relationship between calorie intake and infertility is not linear. Although humans have highly adaptable diets, it seems probable that extreme environmental contexts would have produced sufficient calorie deficit to impact upon fertility in the past. Such situations were likely temporary but may have occurred on a cyclic or random basis in response to various environmental conditions or activities, such as warfare (for example, destruction of crops during raids as discussed for the Marquesas Islands by Molle & Marolleau, 2022).

Energetic expenditure has a strong recognized effect on fertility. Various studies of unmechanized labor (Jasienska & Ellison, 1998; Ruiu & Breschi, 2020) and the introduction of labor-saving technologies suggest that a reduction in energetic expenditure (though behavioral changes may also be implicated) may increase fertility in populations with minimal or no known use of contraceptives (Gibson & Mace, 2002; Kramer & McMillan, 2006). Similarly, age at menarche is influenced by energetic expenditure, with females involved in manual labor typically having a later age at menarche (Udry & Cliquet, 1982). Late age at menarche can be associated with clinical subfertility and infertility, but the increase in risk compared with earlier menarche counterparts is typically quite low at < 0.5% (Guldbrandsen et al., 2014; Komura et al., 1992; McKibben & Poston, 2003). Early age at menarche may reduce the age at first birth and increase the reproductive lifespan, yet the potential for increased fertility is often mediated by social aspects (Sandler et al., 1984). Menarche can trigger a range of social processes contributing to fertility, including perception (by others) and identification (by self) as sexually mature, which then starts a series of culturally predicated events, such as first sexual intercourse, marriage, and first pregnancy (though not necessarily in that order for all cultures) (Udry & Cliquet, 1982).

There is a complex relationship between energetic inputs and outputs, and their interactions with one another and other mediating factors should be considered when examining past population dynamics. That being said, the technologies and physical exertion associated with different subsistence strategies may be a valuable and accessible factor in the study of past population fertility. Energy expenditure among forager-horticulturalists is typically high (Christopher et al., 2019); however, it seems reasonable to suggest that many early farming activities also came with a high energy cost in the absence of modern mechanization. For example, archaeological evidence from the Neolithic site of Con Co Ngua in Vietnam suggests that substantial physical input and expense was associated with the transition to agriculture, indicated by skeletal remains exhibiting a range of injuries likely incurred during interactions with water buffalo (Scott et al., 2019). Further support for this is reported by Macintosh et al. (2017), who found a substantial increase in robusticity of the humerus (upper arm) in farming women over several thousand years following the introduction of agriculture, which they argued was indicative of participation in significant manual labor. As such, availability of energetic resources, subsistence strategy, and associated energy expenditure may influence fertility, and archaeological sites typically contain vestiges of these activities. Some of the primary examples are isotope analysis of diet, archaeological evidence of food production and storage, evidence of land clearing, and zooarchaeological remains.

Other physiological variables have been found to influence fertility. Low birth weight of the mother has been found to influence the size and function of reproductive organs and subsequently may decrease fertility. However, the same mothers are more likely to have children at a younger age, which may serve to increase it (Coall et al., 2016; deKeyser et al., 2012). Sexually transmitted disease is widely reported to influence both male and female fertility on an individual basis, but quantifying the influence has proven challenging. Relevant diseases include gonorrhea, chlamydia, and syphilis (Tsevat et al., 2017). Via a range of causal pathways including inflammation, tubal defects, and miscarriage, these diseases can significantly impact on fertility (Genç & Ledger, 2000; Menon et al., 2015; Reekie et al., 2019).

Paleopathological (based on skeletal lesions, e.g., Vlok et al., 2020) and paleogenetic (using ancient DNA; e.g., Giffin et al., 2020; Guedes et al., 2018) analyses provide insights into the origins and spread of disease, including those that are sexually transmitted, such as treponemal infection. Furthermore, historical accounts can be used to contrast the antiquity (or lack thereof) diseases and whether they were introduced through migration or invasion (see Baker et al., 2020, for an in-depth discussion of the spread and evolution of treponemal disease). Given known transmission, disease progression, and mortality rates, as is the case for syphilis, it may even be possible to quantitatively estimate the influence of morbidity on fertility in the past. Both paleopathology and paleogenetics provide promising avenues for integrating the influence of disease on fertility in studies of past populations.

Common environmental factors influencing fertility include rainfall and temperature. The seasonal nature of births, as a globally observed phenomenon, has sparked substantial interest in climate–fertility interactions, both biological and social aspects. Summer heat appears to result in increased contraceptive use, and decreased conceptions and fertility (Barreca, 2017; Lam & Miron, 1994; Sellers & Gray, 2019). Cold weather has also been shown to have a complex relationship with fertility: in a study by Lam and Miron (1996), extreme cold appeared to have a negligible effect; however, cold and dark seasons in various parts of the world are associated with decreased vitamin D and decreased sexual hormone concentrations, which may reduce fertility (Rojansky et al., 1992). The relationship between births and seasonality is notably variable across the globe, but a combination of heat impacts on sperm count and motility and, in particular, reduced frequency of sexual activity are suspected mechanisms (Lam & Miron, 1994). Delays in monsoon onset in Indonesia have been reported to result in a reduction in contraceptive use among wealthy women, though the causes behind this are unclear, and a corresponding absence of change in births may indicate an increased risk of miscarriage during episodes of environmental stress (Sellers & Gray, 2019).

For the purpose of evaluating past environments and environmental influences, paleoclimate data can be obtained from a range of sources, such as ice and sediment cores, tree rings, lake and sea sediments, and corals, giving insights into both temperature and precipitation. Reconstructions of paleoclimate have been undertaken for most of the globe, including large regional studies by Neukom and Gergis (2012) for the Southern Hemisphere spanning the past 2000 years, and research reported in the edited volume by Emeis and Dawson (2003), in which contributors estimated the paleoclimate record for Europe and the North Atlantic throughout the Holocene. Climatic events, such as El Niño activity (e.g., Martin et al., 1993), and environmental responses, such as changing sea levels (e.g., Nunn, 2000), have also been explored in the context of paleoclimate evidence. There is, therefore, a wealth of paleoclimate information that can be analyzed in comparison with paleodemographic fertility proxies to understand the effects of the environment. In any case, the complex nature of environment–fertility interactions suggests that exploratory research may be more suitable than hypothesis testing, and that global variability in interactions should be expected.

In some cases, it may prove useful to view the interplay of biological and behavioral fertility responses from an evolutionary perspective. As described by Coall et al. (2016), in poor physiological conditions the body may conserve energy and delay maturation (and reproduction as a consequence) so we may expect to see reduced fertility as a result. However, severe conditions that include exposure to high mortality rates in the prospective mother’s social group may, in contrast, accelerate reproduction and the number of offspring to increase the likelihood of at least one child surviving to adulthood, and this may collectively contribute to repopulation (Coall et al., 2016). These and other adaptive responses to unfavorable environmental and psychosocial conditions (e.g., Amir et al., 2016) should be hypothesized cautiously for past populations since such relationships are very dynamic and are not robustly supported across all studies (Kyweluk et al., 2018; Sear, 2020). Life history theory may help to explain adaptations to energetic stress, availability of resources, and exposure to risks and mortality since plasticity allows individuals to change strategies as conditions change.

## Mortality and Anthropogenic and Natural Disasters

Environmental risk and exposure to mortality can come in many forms. Mortality and risk may be driven by disease, famine, warfare, and natural disasters, and notably there is often a relationship between them. But as is true of environmental and energetic resources and stress, the way in which mortality and risk environment interact with fertility is not always straightforward. Fertility may increase with higher mortality rates (Caudell & Quinlan, 2012), while high fertility and high mortality (particularly female mortality) can be predictive of later age of menarche, possibly because of girls adapting their maturation rate in response to observed unfavorable conditions in their environment (Šaffa et al., 2019). Child mortality may specifically be linked to fertility, with decreasing child mortality being associated with decreased fertility and vice versa, due to the additional parental investment and resources available for fewer children (Azarnert, 2006).

Mortality can be surprisingly difficult to estimate for past populations. The only available evidence for mortality in the deep past (before record-keeping, or where records have not survived) is skeletal remains and the ages at which the individuals died, but understanding mortality is complicated by the relatively greater influence of fertility than mortality on the age-at-death distribution. This is an outcome of a highly fertile population having a larger number of infants and young children who are vulnerable to a variety of causes of death, but particularly from disease and malnutrition. Therefore, the age-at-death distributions for highly fertile populations typically have an overrepresentation of children under 5 years of age as a proportion of all deaths (see McFadden et al., 2022). Notwithstanding, unusual frequencies of death in certain age groups (particularly robust ones, such as older children and young adults) may indicate increased mortality in a population, and there are tools for analyzing age-at-death data to estimate the maternal mortality rate (McFadden & Oxenham, 2019; McFadden et al., 2020). Further, paleopathological evidence may be indicative of the disease burden within the population and its likely influence on mortality. In addition to archaeological evidence, there are often ethnographic and oral history accounts of major mortality events.

Anthropogenic disasters, such as armed conflict and warfare, may increase mortality and reduce fertility during their occurrence due to the significant disruption and stress they cause, but often a subsequent rise in fertility is observed (Agadjanian & Prata, 2002; Blanc, 2004; Heuveline & Poch, 2007; Urdal & Che, 2013). Environmental instability and stress (as well as decreased healthcare and education, though these are not relevant to the present study) are implicated in this relationship (Urdal & Che, 2013). Natural disasters too have a range of risk, stress, and mortality impacts that may influence fertility. Hurricanes and earthquakes have an immediate suppressive effect on fertility followed by a stimulative effect, which may last several years (Davis, 2017; Nandi et al., 2018). Broadly speaking, there is a substantial body of evidence to support increased fertility following natural disasters of varying mortality impacts, suggesting mortality is not the sole factor in the increased fertility (Behrman & Weitzman, 2016; Finlay, 2009; Nobles et al., 2015). Notwithstanding, mortality can play a more specific role in fertility strategies, such as when a couple experiences the loss of a child, or when those without children prior to a disaster actively seek to rebuild the community by starting a family at a younger age than previously intended (Nobles et al., 2015). As such, reproductive intentions appear to shift in response to such events. Though complex, these effects may be differentiated into period effects and cohort effects (Hobcraft et al., 1982), the former describing events such as warfare or natural disasters that impact all individuals broadly, and the latter being those which affect individuals differently depending on certain attributes, such as those who personally experience loss of life.

Both archaeological and ethnohistoric sources often capture the timing of anthropogenic and natural disasters and can allude to the scale. Archaeological evidence of fortification, skeletal evidence of trauma, and military burials (often, but not always, indicated by an overrepresentation of young to middle-aged men) may provide a proxy for the frequency and intensity of conflict. Natural disasters often have significant geological and environmental impacts that may be detected through archaeological analyses. Such events may also be passed on through oral traditions. A sixteenth-century tsunami that struck an island in Kiribati is recounted by the local traditional storyteller and well known to the population of the island; the story is corroborated by geological data (Terry et al., 2021). This is not always the case, however, as Janif et al. (2016) found recollections of natural disasters in a population in Fiji were restricted to those that occurred in the lifetime of the individuals describing them. A combination of archaeological and ethnohistoric approaches may best inform us about the occurrence and magnitude of natural disasters, and the subsequent impacts on fertility.

## Culture and Agency

Physiological, environmental, and mortality factors that interact with fertility almost all have interactions with culture too. The variables and mechanisms discussed in the preceding sections may restrict or increase the potential for fertility, but frequently cultural and social norms and individual agency will intervene or facilitate adaptation. Culture and agency, both of which encompass sexual and marriage behaviors, kin relationships and care, and contraceptive use, have a substantial influence on fertility, a relationship which has been particularly neglected in studies of past populations. This is not without cause: accessing information from past populations on cultural and social norms, kin care, agency, and contraception can be very challenging. However, there are opportunities to make cautious inferences from oral histories on customs and traditions, bioanthropological care models, and hypothetical agent behaviors.

Age at first marriage is often related to age at menarche, reproductive lifespan, and fertility rate. Relatively later age at first marriage appears to result in decreased fertility, even in cases where age should not biologically influence fertility (Harwood-Lejeune, 2001; Maitra, 2004; Nanda, 2005; Solanke, 2015). Long-term, broad-scale trends in Japan, South Korea, and Taiwan have shown an increase in mean age at first marriage and an overall decrease in marriage and a corresponding decrease in fertility (Jones & Gubhaju, 2009), while a study by Varea (1993) in Morocco found that women who married earlier also ceased childbearing earlier, and women who married later had children up to the cessation of their reproductive lifespan. These global trends suggest that most births occur within marriage and that later age at first marriage can shorten the reproductive lifespan; however, there are exceptions to this pattern, and contraceptive practices may play an important role.

In terms of family structure, the prevalent type of marriage in a population, monogamous or polygamous, is not in itself a consistent predictor of fertility (Dodoo, 1998; Sear et al., 2002; Sichona, 1993). Sear et al. (2002) noted that polygyny, the marriage of a man to more than one woman, is highly variable in practice and in implications, which may explain varied findings (Josephson, 2002; Sueyoshi & Ohtsuka, 2003). Polyandry, the marriage of a woman to more than one man, has been associated with decreased fertility at the community level in some cases but also has diverse effects (Johnson & Elmi, 1989; Johnson & Zhang, 1991; Ross, 1984). When a wider view of the family structure is taken, alloparenting has been found to play an important role in fertility intentions and resulting rates (Sear et al., 2002; Tanskanen & Rotkirch, 2014; Tanskanen et al., 2014). Šaffa et al. (2019) posited that the perceived risk of not having such care available (due to mortality of adult females) may reduce fertility, but any such relationship would be highly context specific. These complex and variable relationships between marriage practices, alloparenting, and fertility would need to be investigated in a fine-grained manner, which will very rarely be feasible given the nature of past population data. Nonetheless, it is important to theorize how such factors may have shaped reproductive strategies in the past, and at the very least such factors should be acknowledged as having a potential, albeit no longer visible, effect.

Ethnohistoric accounts and oral traditions provide the greatest source of information on these facets of life but notably may be biased, particularly where ethnohistoric accounts come from an invading population, or where oral traditions may have a political motivation (although the political motivations may themselves be of interest; see Sheppard et al., 2004, for discussion). An example of one such resource is *Marquesan Sexual Behaviours* by Suggs (1966), which provides a detailed account of sexual and marriage norms and customs from interview data and include intergenerational perspectives, which are extrapolated to explore how such norms and customs may have manifested prior to European contact. There are also some extensive oral histories which report genealogies, including marriages, for many preceding generations (e.g., Bulbeck, 2006). Evidence for these types of cultural norms and customs may be more widely spread through ethnohistories, but cautious interpretations of such accounts and integration of oral traditions may help to illuminate this area.

Perhaps the most important variable in fertility is agency, but it is immeasurable in the past. However, we may be able to examine some of the conditions that may factor into decision-making (many of which have already been touched on in this discussion). Fertility-awareness-based methods (FABM) of contraception, such as periodic abstinence, are still used by many today and were likely used in the past, though their true antiquity is not known. A study in central Africa found 12% of women were using traditional contraceptives (Rossier & Corker, 2017), including pre-ejaculation withdrawal, but the efficacy of these and other indigenous methods (such as herbs and waist bands) is unknown (Moroole et al., 2020). Modern contraceptives can have a powerful influence on fertility and female empowerment (e.g., Akim & Kembo, 2011; Craig et al., 2016), but uptake is variable and alludes to relationships with agency (desirable high fertility), social and cultural norms (e.g., the influence of religion), and unpleasant side effects (Konje & Ladipo, 1999). Interbirth intervals are impacted by the length of breastfeeding (which may be influenced by subsistence strategy and the availability of suitable weaning foods) and traditions or taboos relating to postpartum abstinence (Campbell & Wood, 1988). Importantly, interbirth interval intersects with a range of sociocultural practices, including marriage and alloparenting.

It is very difficult to make any claims as to the use of contraceptive and abstinence practices in past populations. Even where there is tangible evidence, such as the presence of flora with contraceptive properties, we may never know if they were consumed or the intention behind their consumption (e.g., the plant silphion, which was harvested to extinction in ancient Greece and possesses contraceptive properties; Riddle & Estes, 1992). Given the availability of methods that do not require chemical nor technological mechanisms, it seems plausible that people did use contraceptive practices in the past. Historical and ethnographic accounts support the likelihood of this. For example, Jolly (2001) describes the tradition of postpartum abstinence in Vanuatu to space babies like taro in the garden, as well as the use of abstinence and indigenous contraceptives more generally. Pre-ejaculation withdrawal and menstrual cycle monitoring combined with timed abstinence seem likely candidates for agent control over fertility in the past. We may not be able to estimate the influence of contraceptive practices on fertility in the past, but we should not deny its potential either. Through background determinants, abstinence and contraceptive use may be analyzed in the proposed model as latent variables.

In a study modeling the impacts of status on small-scale societies, inequality and social competition (in relation to relative rank) served to decrease mean fertility (Shenk et al., 2016). The status–fertility relationship has seemingly reversed over time, from high-status individuals having a larger number of children than those of low status to high-status individuals having fewer children than those of low status (Skirbekk, 2008). Historical research indicates that in the United Kingdom high socioeconomic status was associated with having more children up until the 1700s (Boberg-Fazlic et al., 2011), though this relationship appears to be variable (Dribe et al., 2014; Snopkowski et al., 2016).

The social status of an individual can be challenging to determine from their remains since we do not have knowledge of the intentions of the people who buried them, nor the true meaning of burial goods and practices. However, increasing social stratification and complexity may be indicated by relative differences in a range of indicators within a population, such as diet and nutrition (analyzed through isotopes, recent examples including Colleter et al., 2019, and Wang et al., 2020), disease and paleopathology (e.g., Zuckerman et al., 2022), evolution of kinship groups and the relationship between family and power dynamics (Johnson & Paul, 2016), and differential burial contexts (Härke, 2014). Indeed, even the overall size and density of villages can indicate increasing social and political complexity, which often leads to stratification (e.g., Higham, 2015). This evidence may be used to gain insights into the sociopolitical context and the potential impacts this may have on fertility.

Having reviewed the literature on fertility interactions with a range of environmental, sociocultural, biological, and demographic variables, this paper now turns to proposing a new approach to the analysis of such relationships in past populations. The review has highlighted the variable and complex nature of fertility, reinforcing the need for well-considered and explicitly articulated causal pathways in research on past populations.

## Materials and Methods

Using the empirical understanding of fertility interactions, and the identification of variables which can be observed or estimated in the past, I have developed a generalized path diagram to visualize such associations. The diagram sets out logical pathways between variables and the outcome of fertility. This model is intended to provide foundations only and must be adapted for application to any given past population based on knowledge (or hypotheses) of the local cultural, environmental, and biological conditions and their likely fertility interactions. The purpose of the model is to guide practical applications and as such has a pragmatic focus. Some of the variables discussed in this paper are excluded from the path diagram due to the inability to assess, estimate, or model their influence in any past population. However, variables that are observable in some cases but not others are prime for inclusion: the model may be able to predict these variables based on others as the number of applied studies increases (in a similar way to its intended use for populations without demographic proxies).

The exact scale of localized approaches will vary on a case-by-case basis, but consideration should be given to how living descendants believe population boundaries were defined in the past, and evidence from linguistics, ethnography, and culture, to name only a few. A population may be treated as the people who populated a temporally and spatially defined zone, with the temporospatial delineation being based on indicators of shared characteristics. One of the challenges in defining populations is that they often do not occupy an easily delineated space; shared characteristics may be temporally continuous or spatially mobile. Therefore, our investigations into past populations will often tend to capture more than one population or conversely may impose a temporal delineation that is not recognized by living members of a population. These limitations may be unavoidable in some cases but should always be acknowledged.

Though they have been segregated by separate headings in the literature review, I have alluded to the fluidity between these broad categories—energetic, physiological, and environmental resources and stress; mortality and anthropogenic and natural disasters; and culture and agency—of factors that may interact with fertility. It is important to reiterate that among these groupings there are interrelationships which mean that few (or possibly none) of these variables can be treated as wholly independent. We would expect subsistence strategy and subsequently energetic balance to be heavily influenced by the local environment, and in turn subsistence strategy has consequences for sociocultural practices and social hierarchy. For example, a study by BenYishay et al. (2017) found reef density was predictive of female land inheritance in the Solomon Islands, representing adaptation of inheritance rules to ecological conditions. Conversely, pastoralist populations are far more commonly patrilineal (Holden & Mace, 2003; Shenk et al., 2010). The most preferable approach to dealing with this is to accurately set out interrelationships in the path diagram. However, given data available for past populations are often limited in many respects, there will be circumstances where this is not possible. Where that is the case, intercorrelated variables may become hidden within the model and it may be necessary for researchers to discuss all intercorrelated factors (based on the literature) as potentially having an effect in the causal pathway.

**Figure 1.**
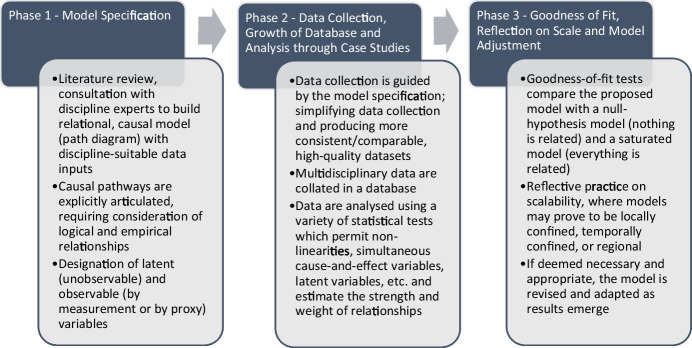
Flow chart of phases in structural equation model approach.

To demonstrate how to operationalize this approach, the following flow chart outlines the process of progressing from a path diagram to structural equation modeling (Fig. 1). This paper focusses overwhelmingly on the literature that underpins phase 1 of the approach, which is to specify the model based on expectations of the relationships between variables, and the data inputs. In application, phase 1 should also include selection of the background determinants and proximate determinants (preferably guided by Wood, 1994) based on the evidence that is known to be available for a case study. Phase 2 involves rigorous data collection, guided by the specified model, and phase 3 compares goodness of fit across comparative models and adjusts the model if required.

### A Simulated Example of Structural Equation Modeling for Past Fertility Interactions

Though intended largely as a guideline, the utility of this approach must be demonstrated if it is to be successfully adopted. The resource-intensive nature of such a project should not be underestimated: the approach advocated for in this paper requires careful and thoughtful consideration of causal pathways within a specific context (ideally with multidisciplinary contributions to ensure an accurate model is constructed to accommodate transdisciplinary inputs) and subsequently requires the collection of large volumes of data to ensure a comprehensive and testable model. The benefits of the proposed approach are only truly realized through a project of substantial scale. However, to demonstrate the potentially powerful insights that may be gained through doing so, a subset of the path diagram was used to guide a structural equation model analysis as an example exercise. Due to the greater availability and accessibility of environmental data, an environmental model was used to facilitate a feasible example case study. The model includes a number of environmental variables that may be expected to interact with fertility based on modern observations. The example study was localized to the Pacific Islands since good data are available for paleoclimate variables and paleodemographic fluctuations have been previously estimated (McFadden et al., 2021). Additionally, the use of an island model (see Terrell, 2020 and Leppard et al., 2021 for robust discussion) hypothetically assists in streamlining this demonstration exercise. The exclusion of energetic and sociocultural factors from this model is not, in any way, an endorsement of environmentally deterministic models. Within the scope of this paper, the selection of environmental variables is purely driven by feasibility and the inherent complexities of investigating energetic and sociocultural factors in past populations. This case study sets out just one aspect of a holistic analysis of past fertility. Further, the use of regional data was driven by data availability, and future studies should use a more localized approach.

Fertility proxy data, arable land, total land, and island type were obtained from the study by McFadden et al. (2021) examining population increase in the Pacific Islands. In this study, fertility was estimated based on the age-at-death distribution of burials, which may be used as a proxy for population increase (McFadden & Oxenham, 2018b). Island types were defined as volcanic high islands, island arcs, or coral atolls (other island types exist, but data pertained to these types only). Further data inputs of sea level, mean temperature, and El Niño activity were obtained from Nunn and Britton (2001) as estimates based on a graphical representation of the collated data, in the absence of reported raw data. The individual sources of the data for this graph were also consulted, but they too failed to report raw data. Nonetheless, the graphical representation in Nunn and Britton (2001) affords a reasonable degree of accuracy, sufficient for this example exercise. These further data inputs are spatially generalized but temporally specific, reflecting fluctuations in environmental conditions throughout the Pacific region at reasonably precise time points. This was considered acceptable for the purpose of demonstrating the proposed approach in operation; however, the broad geographic span and low resolution of these data limit the true interpretative power of the example study, and a more thorough and localized study should be undertaken for investigatory purposes. Missing values (21 of a total of 161 datum points) were estimated using regression analysis, where cases with complete data are used to develop regression equations to predict the missing data. The example model was analyzed, comprising data from 23 populations across seven variables, using SPSS Amos (2021). Structural equation modeling can use a multitude of statistical tests to inform the strength of relationships: in this analysis, correlation was used to test relationship strength between variables and fertility, and goodness of fit was evaluated using chi-square.

## Results

### Foundational Path Diagram

**Fig. 2:**
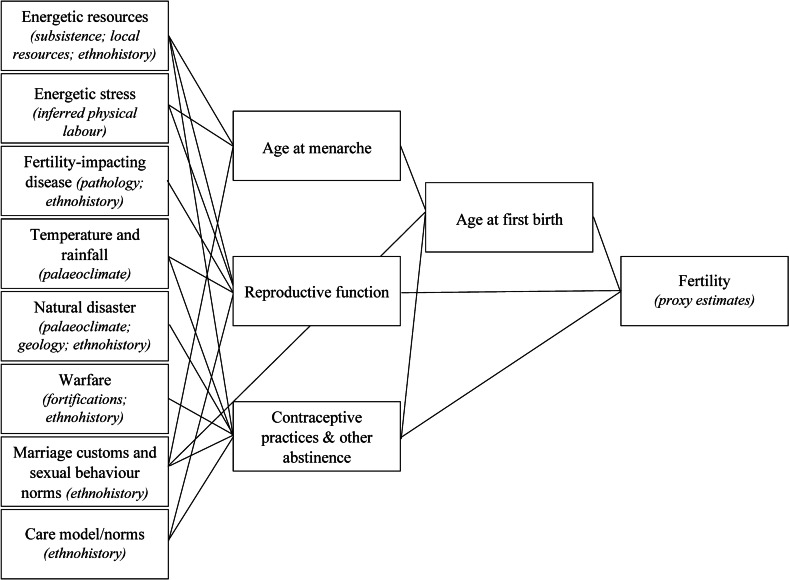
Path diagram of fertility interactions for application to research on past populations. Potential resources in italics

The generalized path diagram (Fig. 2) was developed based on modern literature on fertility interactions and archaeological, historical, and ethnographic literature that informs how these interactions may be assessed in the past.

### Specified Environmental Model and Structural Equation Modeling Exercise

**Fig. 3:**
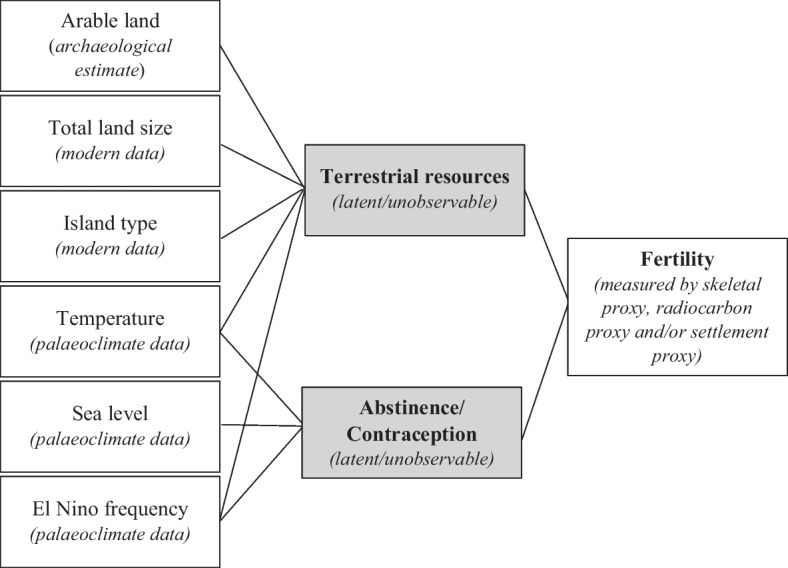
Example path diagram for an environmental model of fertility interactions. Data source/type in italics

Figure 3 provides an example of a simplified path diagram containing a number of environmental variables that interact with fertility based on modern observations. The model reflects solely the environmental component of fertility interacts and should be incorporated into a more holistic model for application to populations in the Pacific Islands. The diagram identifies the nature of the source data that may be used to analyze the model. As previously discussed, modern observations suggest that variation in a range of climate variables may influence reproductive intentions. In this model, climate variables (temperature, sea level, and El Niño frequency) are anticipated to impact patterns of abstinence or contraceptive use. Arable land, land size, island type, temperature, and El Niño frequency may all impact the availability of terrestrial resources through such mechanisms as soil fertility, available space for horticulture, and cyclic effects on resource availability and quality. Terrestrial resources form only one part of diet composition and may influence fertility through energetic balance (e.g., manual harvesting or processing activities). The use of terrestrial and marine resources is also heavily influenced by sociocultural practices; this is true for abstinence and contraceptive use also. As such, it is clear the model provided here is only a snapshot, for the purpose of method demonstration, of these complex and multifaceted interactions.

**Fig. 4:**
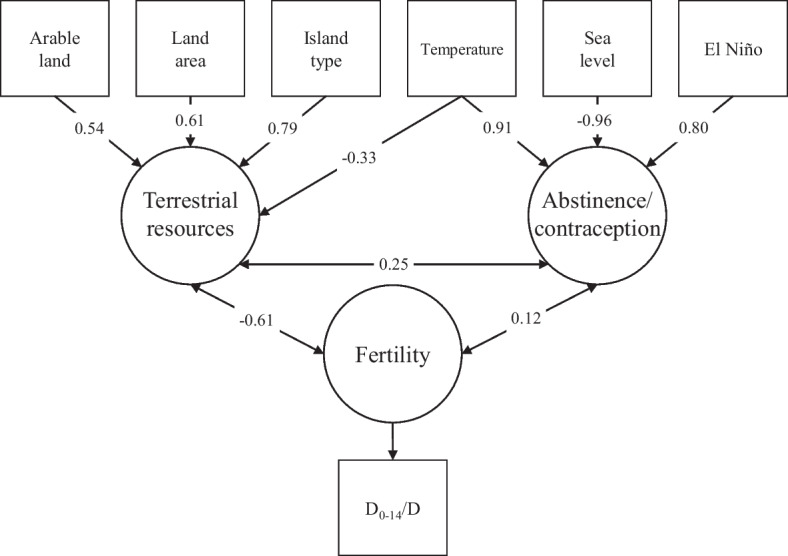
Specified structural equation model for environment–fertility interactions with correlations

This model was analyzed in SPSS Amos Version 28 (2021, https://www.ibm.com/support/pages/ibm-spss-amos-28-documentation) using data derived from McFadden et al. (2021) and Nunn and Britton (2001), including estimates from the latter publication (in the absence of published raw data) and simulated data where values were missing (21 cases, estimated by regression analysis). When compared with the null hypothesis model (nothing is related) and the saturated model (everything is related), the specified model demonstrated a stronger relationship with fertility (indicated by the D_0–14_/D proxy from McFadden et al., 2021). Figure 4 visualizes the specified model and correlations between variables, and Table 1 reports the model comparison. In structural equation modeling, a nonsignificant outcome indicates the model fits the data. In this analysis, the specified model outperformed the saturated and independence models. However, this is only a simplified, environmental model for demonstration purposes, applied to one regional example, and does not preclude the inclusion of further variables, nor alternate models, from having a better overall fit. Indeed, the model set out in Figs. 3 and 4 is restricted and in some cases is based on low-resolution data, so we should expect the addition of sociocultural, biological, demographic, and further environmental variables and the inclusion of high-resolution data to improve the goodness of fit.

**Table 1:** Comparison of the specified model with saturated (everything is related) and independence (nothing is related) models

Model	Number of distinct parameters	χ^2^	DF	*p*	χ^2^/DF
Specified model	20	11.79	8	.16	1.47
Saturated model	28	.00	0	NA	NA
Independence model	7	115.27	21	.00	5.49

## Discussion and Conclusions

Though it is clear that myriad factors can influence fertility, it is less clear under what conditions specific factors will do so. Heterogeneous responses highlight the dynamic and complex interplay of variables, suggesting that highly contextualized studies are essential to understanding population dynamics both now and in the past. Identifying, analyzing, and comparing patterns in fertility interactions from the ground up may assist us in identifying the scale of such patterns as well as improving our understanding of the diversity of populations, their contexts and experiences. Concepts from a range of disciplines, particularly behavioral ecology and life history theory (see Page & French, 2020, for further discussion), are essential to the interpretative framework within which we examine the complexity of interactions and seeming contradictions. In this paper, I have identified and discussed a range of variables that potentially interact with fertility whose past values may be estimated or measured. From this, I have developed a foundational path diagram to guide more locally specific models in the future, which use a range of proxy data for estimates of fertility and interacting variables. Structural equation modeling can then be applied using these model specifications. This approach has many benefits for studies of past populations, including the ability to examine the strength of multiple causal pathways in a single analysis, tolerance of nonlinear (dual cause and effect) variables, flexibility to adapt the model based on inputs, scalability of the model from local to regional (and the ability to see whether the strength of relationships is maintained through this process), and accommodation of latent, unobservable variables. The ground-up approach proposed here provides a loosely set out model based on modern empirical evidence. In application, it should be modified to accommodate the available inputs and respond to data from past populations as it is evaluated.

The example analyzed in this paper demonstrates the utility of such an approach. Not only does it offer preliminary insights into the strength of environmental variables influencing fertility in this example study of the Pacific Islands, it also provides the opportunity to examine the individual causal pathways set out in the model. In this analysis, temperature was expected to impact both the availability of terrestrial resources and agent-based practices such as abstinence and contraception; however, based on the correlations, the relationship with the latter was found to be much stronger than that with the former. Nonetheless, terrestrial resources had an unexpectedly stronger and inverse relationship with fertility than abstinence and contraception. This may be artifactual owing to the inclusion of a greater number of variables predictive of terrestrial resources, or it may be a phenomenon to be explained in future, highly contextualized, localized, and more comprehensive (including energetic and sociocultural variables) research. Importantly, the intention of this example study was to demonstrate how to operationalize the generalized path diagram, in this case as a restricted environmental model in the context of the Pacific Islands. This exercise has demonstrated that the approach provides a multilevel understanding of such relationships, as well as a multiscalar method for determining their relevance under localized through to generalized or regional conditions.

Perhaps the most crucial component of this approach is the requirement for logically and clearly articulated causal pathways. In the big data era, theory is waning, and across disciplines many are calling for a shift in research culture (Harford, 2014; Nurse, 2021). The study of past populations has often sought to leverage as much data as possible as a means to overcoming its scarcity or disparity relative to other fields of study. Such research has provided new evidence and findings, but to improve the depth of insights to be gained from these data, careful consideration of empirically demonstrated relationships, theory, and causality are needed. A ground-up approach using path analysis and structural equation modeling may provide one such remedy. Notwithstanding, the accumulation of case studies and associated data can provide false support for models, particularly in this context where the model is restricted by the nature of archaeological data. As such, models should be dynamic and continually adjusted as further modern and archaeological evidence of fertility interactions becomes available, and the inherent limitations of such research should be acknowledged in interpretations.

## Data Availability

The datasets generated during and/or analyzed during the current study are available in the GitHub repository, https://github.com/claremcfadden/example-sem-past-fertility
